# Melanoma Transition Is Frequently Accompanied by a Loss of Cytoglobin Expression in Melanocytes: A Novel Expression Site of Cytoglobin

**DOI:** 10.1371/journal.pone.0094772

**Published:** 2014-04-10

**Authors:** Yoshihiko Fujita, Satoshi Koinuma, Marco A. De Velasco, Jan Bolz, Yosuke Togashi, Masato Terashima, Hidetoshi Hayashi, Takuya Matsuo, Kazuto Nishio

**Affiliations:** 1 Department of Genome Biology, Faculty of Medicine, Kinki University, Osaka-Sayama, Osaka, Japan; 2 Department of Anatomy and Neurobiology, Faculty of Medicine, Kinki University, Osaka-Sayama, Osaka, Japan; University of Connecticut Health Center, United States of America

## Abstract

The tissue distribution and function of hemoglobin or myoglobin are well known; however, a newly found cytoglobin (CYGB), which also belongs to the globin family, remains to be characterized. To assess its expression in human malignancies, we sought to screen a number of cell lines originated from many tissues using northern blotting and real time PCR techniques. Unexpectedly, we found that several, but not all, melanoma cell lines expressed CYGB mRNA and protein at much higher levels than cells of other origins. Melanocytes, the primary origin of melanoma, also expressed CYGB at a high level. To verify these observations, immunostaining and immunoblotting using anti-CYGB antibody were also performed. Bisulfite-modified genomic sequencing revealed that several melanoma cell lines that abrogated CYGB expression were found to be epigenetically regulated by hypermethylation in the promoter region of *CYGB* gene. The RNA interference-mediated knockdown of the CYGB transcript in CYGB expression-positive melanoma cell lines resulted in increased proliferation *in vitro* and *in vivo*. Flow cytometric analysis using 2′-, 7′-dichlorofluorescein diacetate (DCFH-DA), an indicator of reactive oxygen species (ROS), revealed that the cellular ROS level may be involved in the proliferative effect of CYGB. Thus, CYGB appears to play a tumor suppressive role as a ROS regulator, and its epigenetic silencing, as observed in CYGB expression-negative melanoma cell lines, might function as an alternative pathway in the melanocyte-to-melanoma transition.

## Introduction

Hemoglobin and myoglobin are among the best studied and understood of all proteins. These globins are known to be capable of transporting and storing oxygen, thereby sustaining oxidative metabolism in cells. Cytoglobin (CYGB), a new member of the globin family that was identified together with neuroglobin (NGB), is a hexa-coordinated heme protein [1], [2]. Although CYGB is known to exhibit a high intrinsic affinity to oxygen, similar to myoglobin, its physiological function remains to be clarified [3]. CYGB was originally characterized as a 21-kDa heme protein with an enhanced expression level in stellate cells in fibrotic liver and was initially named “stellate cell activation-associated protein” [4]. A role in the cellular response to tissue fibrosis has been suggested by a study in which the overexpression of CYGB provided protection against chemically induced liver fibrosis [5]. A potential role in reactive oxygen species (ROS) detoxification has also been suggested [6]–[8]. A human neuroblastoma cell line transfected with a plasmid DNA containing CYGB cDNA showed enhanced survival after exposure to H_2_O_2_ [6] and significant protection from oxidative DNA damage induced by a singlet oxygen generator [7]. Furthermore, CYGB has been shown to protect rat kidney fibroblasts against oxidative stress under ischemic conditions *in vivo* [8]. However, most functional analyses of CYGB, including the above-mentioned characterizations, have so far been performed using cells with ectopically expressed CYGB.

Cells endogenously enriched in CYGB, if found, would facilitate the functional characterization of CYGB at an endogenous level, rather than an ectopically induced level that could result in an overestimation of function. The distribution of CYGB in normal tissues has been analyzed in detail. In some studies, CYGB appears to be ubiquitously expressed in whole tissue [1], [9], while the other studies have revealed some cell-types that specifically express CYGB [4], [10]. Compared to normal tissues, tumor tissues or cell lines have not been extensively investigated for the presence of CYGB [11]. This lack of study can be partly explained by the absence of the chromosome region 17q25 (which contains the *CYGB* gene) in multiple malignancies [12]. The transcriptional inactivation of promoters of *CYGB* by DNA hypermethylation has also been shown in lung, esophageal and head and neck cancers [13]–[15]. Such transcriptional suppressions, which are frequently observed in many cancer types, suggest that *CYGB* might function as a tumor suppressor gene, making it difficult to discover cancer cell types overexpressing CYGB. Nevertheless, 1) based on the assumption that, similar to other globins, CYGB could have a specific function in limited tissues or cell types, and 2) according to our initial aim to assess the correlation between CYGB loss and the resultant tumor malignancy, we sought to perform an extensive screening for CYGB expression in several cancer cell lines and found that several, but not all, melanoma cell lines highly expressed CYGB.

## Results

### CYGB Is Expressed at High Levels in Some Melanoma Cell Lines

**Figure 1:**
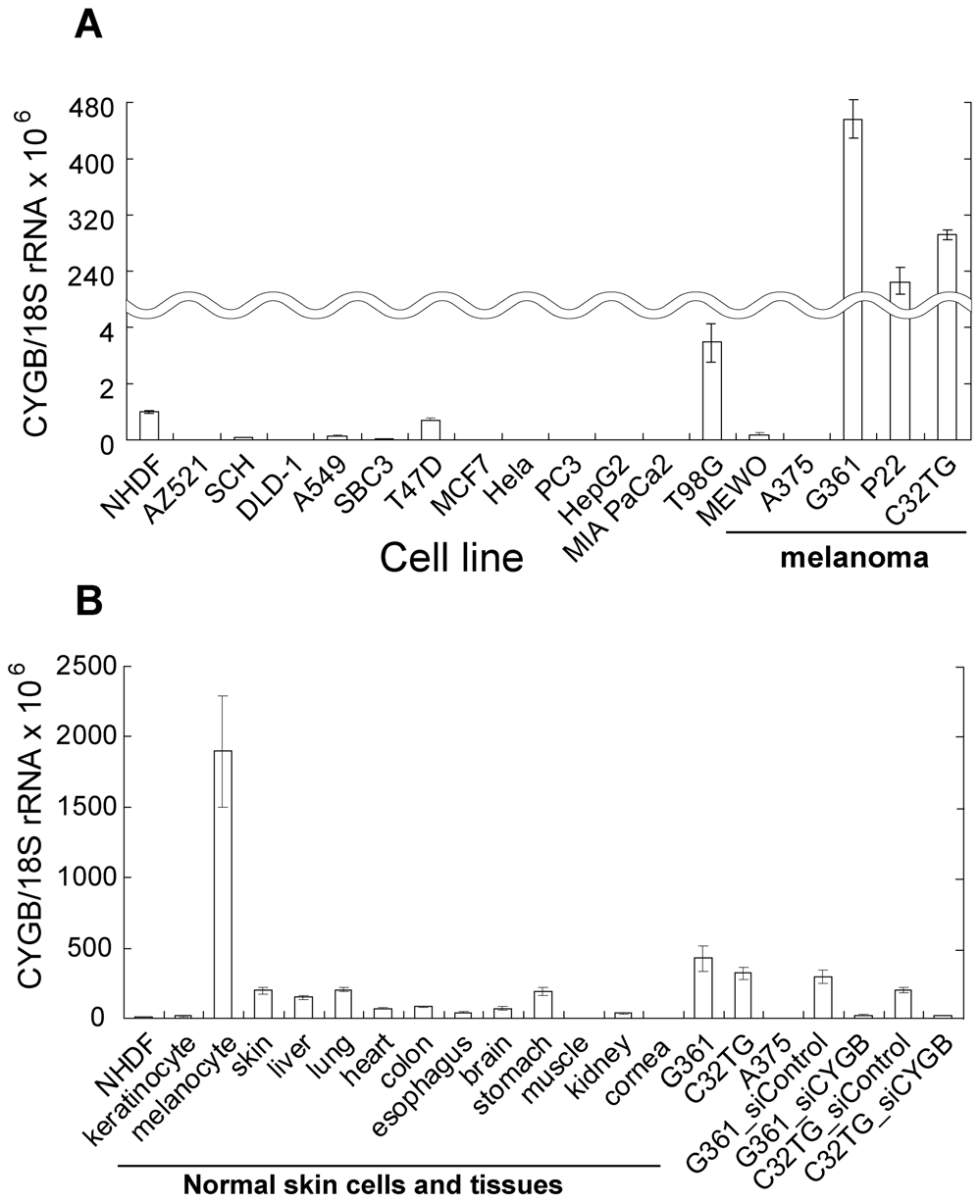
Some melanoma cells and melanocytes express high level of CYGB. Realtime quantitative PCR was performed using a TaqMan probe to detect the *CYGB* mRNA level in **(A)** different human cancer cell lines shown in Table S1 including 5 melanoma cells (A375, Mewo, G361, P22, C32TG) and **(B)** 3 normal cells in skin (NHDF, keratinocytes, melanocytes) and 11 normal tissues (skin to cornea) together with 2 melanoma cell lines (G361, C32TG) and their transfectants with siRNAs (si_Control and si_CYGB). In both **(A)** and **(B)**, the expression of the CYGB transcript was normalized by each 18S rRNA level (n = 3, mean ± SEM) and the normalized CYGB/18S rRNA expression ratio for normal human dermal fibroblasts (NHDF) was set equal to 10^−6^.

To explore the possibility of whether some types of cancer are enriched in CYGB, we screened for CYGB using several cancer cell lines of diverse origins (Table S1). To our surprise, a TaqMan probe-based real-time quantitative PCR revealed that 3 melanoma cell lines (G361, p22, and C32TG) expressed *CYGB* mRNA several hundred-fold more abundantly than the other cell lines that were tested (Fig. 1A). In 2 melanoma cell lines (A375, MEWO), on the other hand, *CYGB* mRNA was detected at much lower levels. To confirm the expression discrepancy among the cell lines, we subjected RNA preparations from each cell to a northern blot analysis (Fig. S1A). The results were in good accordance with those of quantitative PCR analysis, with *CYGB* mRNA being abundantly expressed in the G361, p22, and C32TG cell lines but not detected in the A375 and MEWO (melanomas), A549 (lung cancer), and T47D (breast cancer) cell lines (Fig. S1B). Hypoxic (1% O_2_) or anoxic (0.1%–0.2% O_2_) conditions can significantly up-regulate *CYGB* mRNA in several cell lines as previously reported [11]. Among the non-melanoma cells, T98G cells (glioblastoma) alone produced a slight mRNA signal in response to anoxia for 6 hours (Fig. S1). We next searched a publicly available database for gene expression profiles. The Gene Expression Omnibus database (GEO, http://www.ncbi.nlm.nih.gov/geo) provided microarray datasets for various cancer cell lines. The relative amounts of *CYGB* mRNA calculated for the representative cell lines, including 15 melanoma cell lines, are listed in Table S2. Of these cell lines, a high amount of *CYGB* mRNA was expressed exclusively in melanoma cells, including G361 and C32. As expected, some melanoma cells including A375, SKMEL28 and HS294T formed a group that expressed *CYGB* mRNA levels that were as low as those of non-melanoma cell lines (Tables S2 and S3).

### CYGB Is Overexpressed in Melanocytes

**Figure 2:**
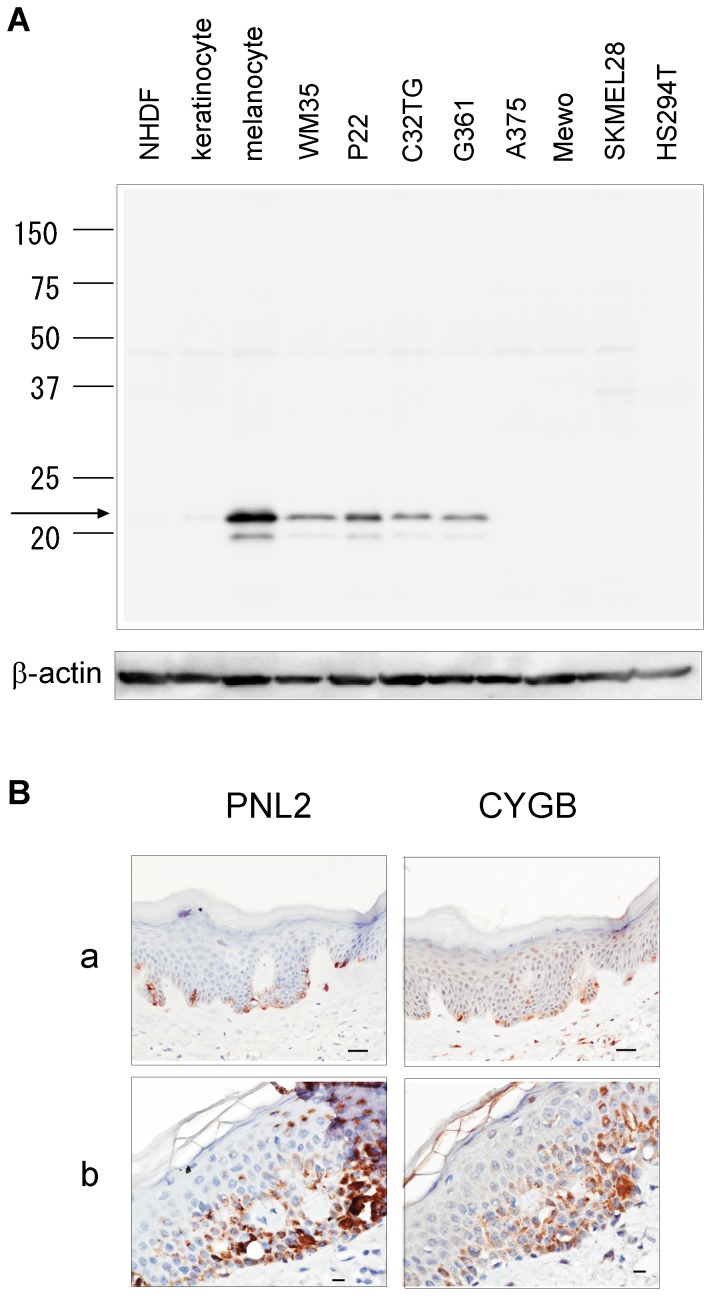
CYGB protein is overexpressed in melanocytes and some of its malignant offspring. **(A)** Immunoblot analysis of CYGB protein (indicated by an arrow) in NHDF, keratinocytes and melanocytes from skin and 8 melanoma cell lines (WM35 to HS294T). The image was obtained using ImageQuant LAS 3000 with an exposure time of 15 sec. The minor band, possibly a degradation product, is observed below the major band, which is prominent in melanocytes. The molecular mass marker (kDa) is given on the left side. β-actin was used as a loading control. **(B)** Immunohistochemical analysis of formalin-fixed, paraffin-embedded human normal skin using PNL2 (melanocyte marker) and CYGB antibodies. Two different regions (a) and (b) stained using each antibody are shown: (a) 4 × magnification, scale bar = 100 μm. (b) 20× magnification, scale bar  = 10 μm.

The unexpected identification of CYGB in melanoma cells prompted us to examine the presence of CYGB in melanocytes, the precursor of melanoma cells. A real-time quantitative PCR assay showed that the expression level of *CYGB* mRNA in melanocytes surpassed those observed in skin and various other normal tissues (Fig. 1B), revealing melanocyte as a prominent cell type that overexpressed CYGB. The level of protein expression in melanocytes was comparable to, or even higher than, the four CYGB expression-positive melanoma cell lines (Fig. 2A). The expression of CYGB in keratinocytes, the main cell type in the epidermis, as well as normal human dermal fibroblasts (NHDF) was only detectable in immunoblot with an increased exposure time (Fig. S2A), suggesting the predominant distribution of CYGB in melanocytes within the skin. Paraffin-embedded sections of normal human skin were then used to examine CYGB expression. Immunoreactivity using an antibody against CYGB showed the same localization at the epidermal basement membrane as that for PNL2 protein, which is often used as a marker for melanocytes, demonstrating that CYGB is highly enriched in melanocytes (Fig. 2B).

The intracellular localization of CYGB has been estimated using immunohistochemistry at the tissue level [4], [10], [16] or using a fluorescent signal from GFP-fused CYGB forcibly expressed in cells [7], [10]. These studies have revealed that CYGB is localized in the cytoplasm of fibroblasts and their derivatives [10], while it is also detected in the nucleus in neurons, various epithelial cells, hepatocytes and connective tissue cells [16], [17]. Melanocytes or G361 cells that endogenously express high amounts of CYGB enabled direct immunostaining for localization (Fig. S2B). These cells expressed CYGB in both the cytoplasm and nuclei, but the expression was rather concentrated in the nuclei of the G361 cells, while A375 cells gave only weak signals.

### Epigenetic Silencing of *CYGB* Gene Occurs in Some Melanoma Cell Lines

We searched the database (GSE29359) to determine whether fluctuations in cytoglobin expression levels, as observed between the G361 and A375 cell lines, are common among melanoma patients (Table S4 and Fig. S3). A relatively high expression level of *CYGB* mRNA was apparent in 7 out of 8 normal melanocyte cell lines, but only 14 melanoma tumor tissues out of specimens from 79 melanoma patients (17.7%) reached the same level. These results suggest that most melanomas lose their CYGB expression during the melanocyte-to-melanoma transition.

**Figure 3:**
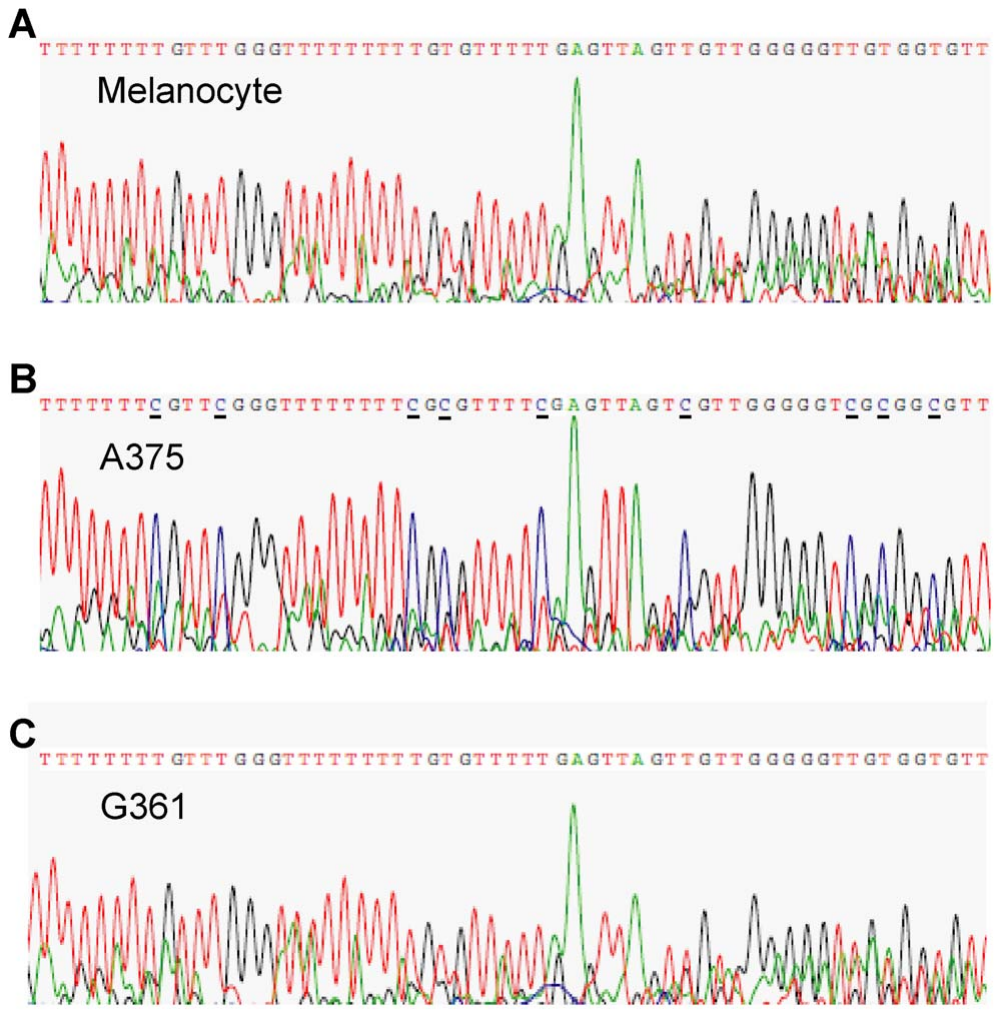
Sequencing histograms for the CpG island of the CYGB promoter region. Of the 24 CpG sites known to be methylated in the CYGB promoter region, 9 sites analyzed for methylation are shown. Cytosines methylated in A375 **(B)** are underlined. The corresponding cytosines are entirely unmethylated in melanocytes **(A)** and G361 **(C)**, resulting in the sequence “TpG” after bisulfite treatment.

Recent methylation-specific PCR assays have provided evidence of higher levels of *CYGB* promoter methylation in lung and esophageal tumors compared with adjacent nonmalignant tissues [13]. We sequenced the promoter region of the *CYGB* gene from melanocytes, G361 cells and A375 cells after bisulfite-modification followed by PCR. Twenty-four CpG sites are known to reside within the *CYGB* promoter region [18], of which 9 are shown in Fig. 3. The results revealed that all 9 CpG sites were methylated in A375 but were totally unmethylated in melanocytes and G361, demonstrating that the transcriptional inactivation of the promoter by DNA hypermethylation occurred in A375 (Fig. 3) and in other melanoma cell lines in which the expression of the *CYGB* gene is down-regulated (Fig. S4).

### CYGB Functions as a Tumor Suppressor Protein in Melanoma Cells

**Figure 4:**
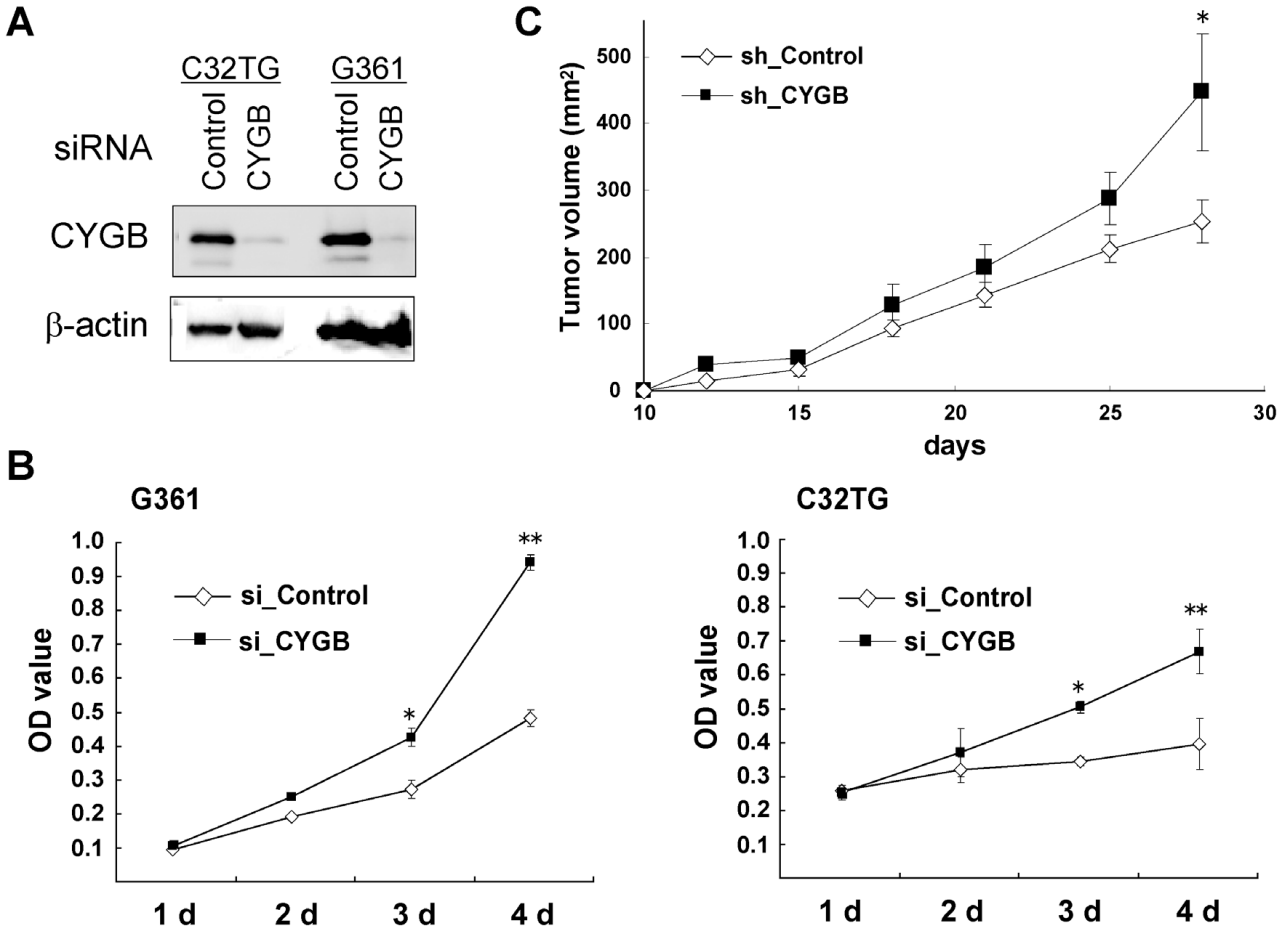
*CYGB*-knocked down melanoma cells increase proliferation. **(A)** Immunoblot data for C32TG and G361 cells transfected with CYGB siRNA or control siRNA. β-actin was used as a loading control. **(B)** Cellular proliferation pattern for G361 and C32TG cells transfected with CYGB siRNA (si_CYGB) and control siRNA (si_Control). The MTT analysis was performed daily (1d to 4d) post-transfection. The value represents the mean from three independent experiments; OD value, 570 nm. bars, SEM. * *P*<0.05, ** *P*<0.01. **(C)** Growth analysis of xenografted G361 tumors in nude mice. G361 cells expressing shRNA against CYGB or control shRNA were subcutaneously implanted into the interscapular region of five female mice. Tumor size was measured at the indicated time points. Bars, SEM. * *P*<0.05.

The epigenetic gene promoter methylation has been well documented for many tumor suppressor genes [19]. *CYGB* has been recently suggested to function as a tumor suppressor gene in non-small cell lung cancer [18] and head and neck squamous cell carcinoma [15]. To clarify whether *CYGB* also functions as a tumor suppressor gene in CYGB-positive melanoma cells, we silenced the *CYGB* gene in G361 and C32TG cells using specific siRNA. Both a quantitative real-time RT-PCR and an immunoblot analyses demonstrated that the CYGB siRNA successfully reduced expression of CYGB in both cell lines but the control siRNA did not (Figs. 1B and 4A). We observed a significantly increased proliferation rate as a result of CYGB knockdown in the CYGB siRNA-treated cells (Fig. 4B), providing strong evidence for the tumor suppressor properties of the *CYGB* gene in melanoma.

To check the validity of these findings *in vivo*, we performed xenograft experiments. In order to maintain a long-term knockdown effect, we first established G361 cells stably expressing short hairpin RNA (shRNA) for CYGB or a nonsilencing control. As expected, a reduced expression of CYGB protein (Fig. S5A) and an increased cell proliferation rate (Fig. S5B) were apparent in cells expressing the CYGB shRNA. Equal numbers of CYGB shRNA- and control shRNA-expressing cells were injected subcutaneously into nude mice and allowed to grow, the tumor sizes were then monitored over time. Both cell lines formed tumors, but the CYGB shRNA xenografts grew significantly faster (Fig. 4C) and had less apoptotic signals (Fig. S5C and D) compared with the control xenografts, again confirming the role of *CYGB* as a tumor suppressor gene.

### CYGB Knockdown Causes an Increase in ROS Level

**Figure 5:**
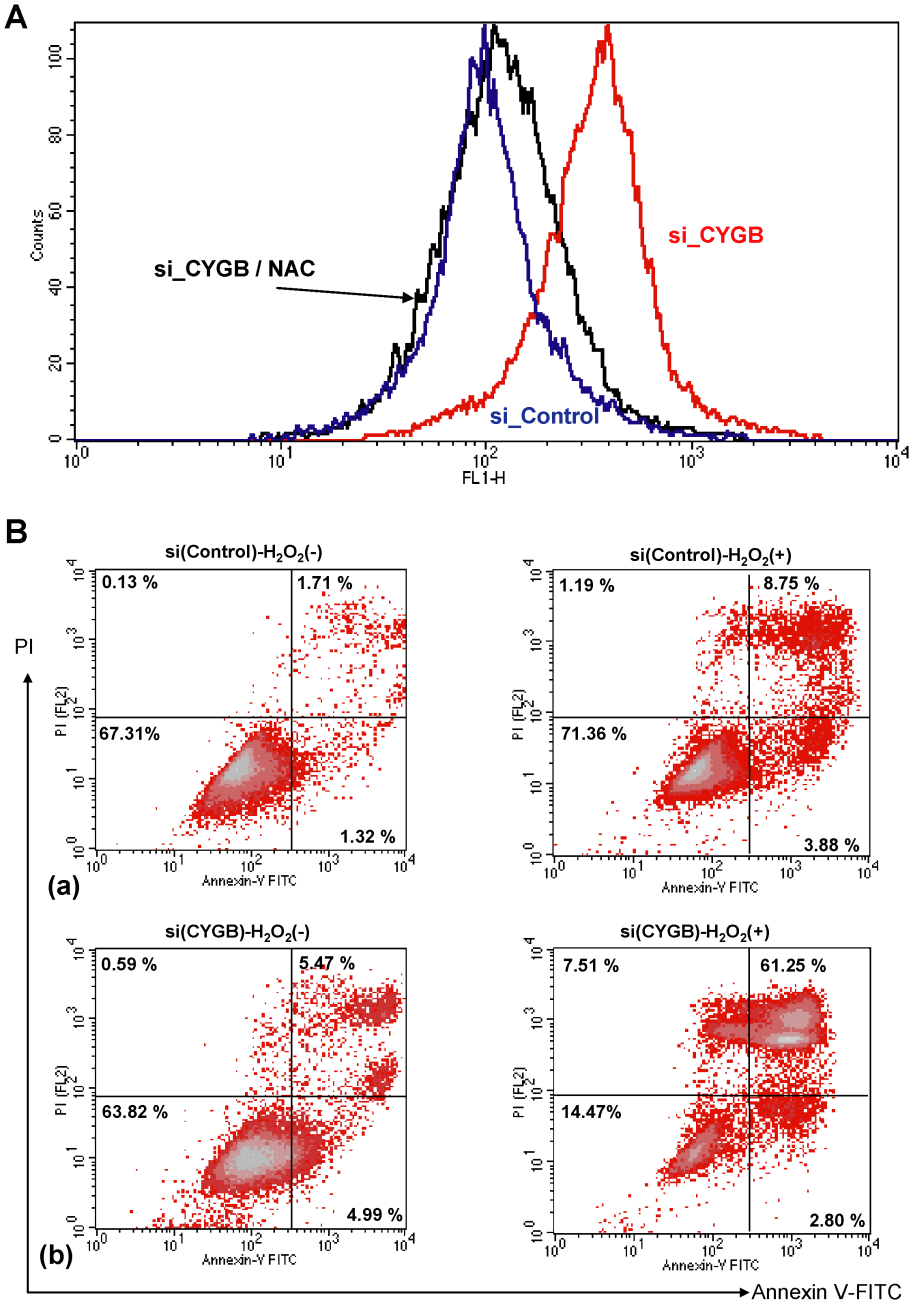
CYGB protects G361 cells from H_2_O_2_-induced cell death. A flow cytometric analysis was performed to determine the ROS level **(A)** and apoptosis **(B)** in G361 cells transfected with a CYGB-siRNA or a control-siRNA. In **(A)**, 2′-, 7′-dichlorofluorescein diacetate (DCFH-DA) was used. CYGB-knocked down G361 cells treated with 3 mM N-acetyl-L-cystein (NAC) were also compared. **(B)** Annexin V and PI staining was done after exposure of the cells to 100 μM H_2_O_2_ for 0 h (−) or 24 h (+). At an early stage of apoptosis the cells bind to Annexin V while still excluding PI. At a late stage of apoptosis they bind to Annexin V and stain brightly with PI. (a) Control-siRNA-transfected, and (b) CYGB-siRNA-transfected G361 cells.

As CYGB has been reported to scavenge ROS when overexpressed in tumor cells [6], [7], CYGB knockdown may raise the cellular ROS level and confer vulnerability that induces cell death. We determined the effect of CYGB knockdown on cellular ROS levels in G361 cells using flow cytometry and the redox-sensitive fluorescent probe 2′-, 7′-dichloroflulorescein diacetate (DCF-DA). CYGB knockdown for 24 hours caused a marked increase in the cellular ROS level. Co-treatment with N-acetyl-L-cystein (NAC) fully reversed the CYGB knockdown-induced increase in ROS (Fig. 5A). Exposure to 100 μM H_2_O_2_ for 24 hours caused a much higher ratio of early and late apoptosis in the CYGB siRNA-transfected G361 cells (61%) compared with that observed for non-treated cells (5.5%) and control cells treated with 100 μM of H_2_O_2_ for 24 h (8.8%) (Fig. 5B).

## Discussion

In the present study, we found that melanocytes are a major cell type that is rich in cytoglobin, similar to how erythrocytes are rich in hemoglobin and myocytes are rich in myoglobin, although the function of CYGB is likely to differ from the latter two globins. Several melanoma cells that escaped from epigenetic regulation were shown to have considerable expression levels of CYGB, retaining their melanocytic character. Their high expression level of CYGB might be attributable to the gene amplification, a process by which the subchromosomal portions of the genome increase in copy number, which has been frequently observed in many human cancers [20] but not in normal cells [21]. The high expression level of cytoglobin in melanocytes is, therefore, unlikely to be due to gene amplification. Rather, some cell-type-specific factors may activate CYGB expression in melanocytes and a part of its malignant offspring, including G361. Microphthalmia-associated transcription factor (MITF) is a protein known to be responsible for the transcription of melanocyte–specific genes. MITF binds to the “CATGTG” consensus sequence found in the promoter region and activates the transcription of melanocyte–specific proteins such as tyrosinase, a melanin-synthesizing enzyme [22]. This motif was not present in the *CYGB* promoter region. Some unknown motifs, through which melanocyte-specific gene promoters are alternatively transactivated, may exist.

In early studies, the distribution of CYGB has been analyzed in normal tissues and CYGB has been found to be uniformly expressed in broad range of tissues [1], [9]. Since these studies analyzed the expression in whole tissue levels, cell-type specific expression within tissues may have been underestimated in whole tissue extracts. In an independent study, Kawada et al [4] had shown enhanced expression of the rat homologue of CYGB in the stellate cells of the fibrotic liver, thus describing it as “stellate cell activation-associated protein” or STAP. CYGB has been also found in distinct cell populations in several tissues such as fibroblasts in connective tissue, chondroblasts in cartilage, osteoblasts in bone and neurons in colon (myenteric plexus) and in brain [10]. The CYGB levels in these cells may be as high as in melanocytes. Indeed, the overexpression of CYGB is detected in some cell lines of neuronal origin (neuroblastomas) (Table S3). Melanocytes are similar to neurons in that they are derived from pluripotent neural crest cells that differentiate into numerous cell lineages [23]. The development of melanocytes and neurons is thought to be controlled by common signaling molecules. The same signaling molecules may also promote the overexpression of CYGB in both cell types.

The nuclear localization of CYGB appears to be rather specific to melanoma cells, compared with melanocytes (Fig. S2B). CYGB has been speculated to play a role in the protection of genomic DNA from oxidative DNA damage; however, as CYGB contains no known nuclear localization signals, the mechanism of nuclear transport and its function in the nucleus remain to be determined.

Within melanocytes, melanins are formed from the successive oxidation of tyrosine, which results in the generation of hydrogen peroxide [24]. This oxidative byproduct, also generated by UV irradiation, is efficiently scavenged within the melanosomes by melanin, which in turn acts as an anti-oxidant [25]. CYGB has also been suggested to play a defensive role against oxidative stress. Human neuroblastoma cells with overexpressed CYGB showed significant protection from oxidative damage induced by H_2_O_2_ [6] or a singlet oxygen generator [7]. Treatment with CYGB siRNA enhanced the cellular ROS levels in fibroblasts from *CYGB* transgenic rat kidney [8]. In melanocytes, highly enriched CYGB may act as a ROS scavenger, similar to melanin.

On the other hand, melanosomes in melanoma cells not only show a dramatically reduced ability to neutralize ROS, but also actively produce excessive amounts of ROS [26]. Thus, the function of the melanosome changes from a ROS scavenger (anti-oxidant) in melanocytes to a ROS producer (pro-oxidant) in melanomas. Melanoma cells produce larger amounts of ROS and exhibit significantly higher levels of oxidative stress, compared with squamous cell carcinoma and basal cell carcinoma in the skin [27] as well as colon, pancreatic, and breast cancer cells [28]. In view of these unique melanoma properties, the elevated production of ROS seems to be a melanoma-specific defect [29], which could be caused by the heavy oxidation of melanin [24] and possibly by CYGB down-regulation for some cell types, as has been shown in the present study.

The amount of ROS produced by melanoma cells, which is within the cellular antioxidant capacity, is rather important for cellular-signaling pathways that induce apoptosis-resistance and cell proliferation [30]. ROS are thought to constitutively activate nuclear factor-kappa B (NF-κB), a transcription factor that is critically involved in cell survival. The activation of NF-κB has been proposed as an event that promotes melanoma tumor progression [31]. On the other hand, high levels of ROS exceeding the cellular antioxidant capacity may have a damaging impact on cells. If CYGB acts as a ROS scavenger in melanoma cells, it may alleviate the high levels of oxidative stress. Several cellular defense mechanisms have also evolved to protect cells from ROS. These mechanisms include repair systems, detoxifying enzymes such as superoxide dismutase (SOD), catalase (CAT), glutathione peroxidase (GPX) and small molecule scavengers such as glutathione (GSH). However, these antioxidant systems appear to be weakened in melanoma patients, leading to the accumulation of ROS, which may promote the cancer process [32]. Recently, Yamaura et al. [33] reported that treatment with siRNA or an inhibitor of NADPH oxidase 4 (Nox 4) decreased ROS production, thereby blocking melanoma cell proliferation. Nox 4 is known to produce superoxide anion (O_2_^−^), which is readily converted into hydrogen peroxide (H_2_O_2_). Yamaura et al. also showed that the overexpression of CAT, a scavenger of ROS (H_2_O_2_), caused a similar effect on melanoma cells. More recently, another group has shown that the scavenging of O_2_^−^ by a specific compound inhibited cell growth, reduced viability, and induced apoptosis in melanoma cells, indicating that O_2_^−^ is important for melanoma survival [34]. CYGB, like other hexacoordinated globins, may scavenge ROS utilizing heme and thiol residues [35] and could well be a target for gene silencing in melanoma. Whether or not CYGB is lost during the melanocyte-to-melanoma transition may affect tumor malignancy. Indeed, tumors without CYGB were more proliferative (Fig. 4B) under oxidative stress (Fig. 5A), a state that is vulnerable to ROS (Fig. 5B).

In conclusion, we have identified melanocyte as the prominent site of CYGB expression that greatly expands our understanding about the evolutionary diversity of the globin family. In addition, present study indicates that CYGB could be a possible candidate biomarker to predict the malignant potential of melanomas. Knowledge of the role of ROS in melanomagenesis and the mechanisms by which CYGB regulates oxidative stress can aid in the development of better antimelanoma therapies. For example, pro-oxidant compounds that target the cellular antioxidant capacity are expected to selectively kill melanoma cells.

## Materials and Methods

### Cell Lines

All cell lines established from human cancers as listed in Table S1 were purchased from ATCC (Manassas, VA) or Japanese Collection of Research Bioresources (Osaka, Japan). These cell lines, together with normal human dermal fibroblasts (NHDF) (Promocell, Heidelberg, Germany) were cultured in DMEM or RPMI medium (Sigma, St Louis, MO) supplemented with 10% heat-inactivated fetal bovine serum (Invitrogen, Carlsbad, CA), and the cell lines were maintained in a 5% CO_2_-humidified atmosphere at 37°C. Human epidermal melanocytes (HEMa-LP) isolated from lightly pigment adult skin were purchased from Invitrogen. The cells were cultured in Medium 254 (Invitrogen) and were maintained in a 5% CO_2_-humidified atmosphere at 37°C.

### RNA Isolation and Quantitative Real-Time RT-PCR

Cells were washed once in ice-cold PBS and RNA was extracted from each sample using the Trizol method (Invitrogen, Carlsbad, CA). Normal human epidermal keratinocytes (NHEK) purchased as a pellet from Promocell (Heidelberg, Germany) were used to isolate the total RNA and protein. Total RNAs from normal tissues were the product of Takara (Ohtsu, Japan), except for the tongue, throat, esophagus and skin tissues, which were obtained from Biochain (San Francisco, CA). One microgram of the total RNA was reverse-transcribed with AMV reverse transcriptase using random primers and oligo (dT) primer. Real-time quantitative PCR was performed using the fluorescent TaqMan methodology and the ABI PRISM 7700 Sequence Detection System (Applied Biosystem, Foster City, CA). Ready to use, predesigned primer and probe sets (Applied Biosystems) for human CYGB (Hs00370478_m1) and housekeeping gene 18S rRNA (Hs99999901_s1) were used according to the manufacturer's guidelines. The relative expression of mRNA was calculated using the comparative Ct method.

### Northern Blot Analysis

Total RNA (10 μg) extracted from each cell was analyzed using the Ambion Northernmax Kit (Life Technologies) according to the manufacturer's guidelines. Hybridization was performed under standard conditions with a digoxigenin-labeled human CYGB cDNA probe. The final washing conditions were 65 °C at 0.1× standard saline citrate (SSC). The blot filter was exposed for 24 h using Kodak XROmat film.

### Immunoblot Analysis

Immunoblot analysis for CYGB protein detection was performed essentially as described previously [36] using rabbit anti-CYGB antibody, a kind gift from Dr. Norifumi Kawada [8], and β-actin antibody (Cell Signaling Technology, Beverly, MA). The images were obtained using ImageQuant LAS 3000 (Fujifilm, Tokyo, Japan).

### Immunohistochemical Analysis

A skin tumor tissue array (T212a) including normal tissue as a control was purchased from Biomax US (Rockville, MD) and was analyzed as described previously [37] using the rabbit anti-CYGB antibody, and a mouse antibody against PNL2 (Cell Marque, Rocklin CA), a highly specific marker for melanoma and melanocyte.

### Immunofluorescence Staining

To detect CYGB expression in melanocyte and melanoma cell lines, cells grown to subconfluence on cover slips were rinsed with PBS and fixed in 4% formaldehyde for 30 min at room temperature (RT). After rinsing several times with PBS, cells were blocked with 10% goat serum for 30 min and were incubated in PBS containing anti-CYGB antibody (1∶200) for 1 hour at RT. Cells were then washed with PBS and incubated in PBS containing 10 μg/mL of Alexa Fluor 488 goat anti-rabbit IgG (Molecular Probes, Eugene, OR). After washing 3 times in PBS, the cover slips were mounted on a slide glass using Vectashield (Vector Laboratories, Burlingame, CA). The images were captured using a fluorescence microscope (IX71; Olympus, Tokyo, Japan).

### *CYGB* Promoter Methylation Analysis

Genomic DNA was extracted from the G361 and A375 cell lines and melanocytes using Blood & Cell Culture DNA Kits (Qiagen, Hilden, Germany). Bisulfite treatment for each sample (0.8 μg) was performed using the CpGenome Turbo Bisulfite Modification Kit (Millipore, Billerica MA). The modified DNA was subjected to PCR amplification using NovaTaq DNA Polymerase (Merk, Darmstadt, Germany) and primers that were designed to exclude the CpG site, thereby rendering the amplification independent of the methylation status. The primer sequences were as follows: 5′-GGGAATTGATTTAAAGTTTAAT-3′ (forward), and 5′-TAACCCCCCAAACCTAA-3′ (Reverse). The DNA sequencing of the amplicon (matching GenBank accession NC_000017.10) was performed in both directions using the BigDye Terminator v3.1 Cycle Sequencing Kit (Applied Biosystems).

### siRNA Transfection

siRNA targeting human CYGB and negative control siRNA were purchased from Qiagen. The targeting sequences for CYGB and control (scrambled sequence) siRNA were 5′-GGA GGA AUC CCU GAC UCA A-3′ and 5′- GAG CAG UCC CAU CGA UAG A -3′; respectively. The transfection methods have been described previously [38].

### Short Hairpin RNAs (shRNAs) Transfection

Targeting sequences used to construct shRNA for human CYGB and nonsilencing control (scrambled sequence) were same as siRNA sequences. They were cloned into an RNAi-Ready pSIREN-RetroQ-ZsGreen vector (Clontech) according to manufacturer's protocol. The stable transfectants of G361 cells expressing these shRNAs were obtained as previously described [38].

### Xenogtrafts in Nude Mice

Assay of tumor in nude mice (BALB/c-nu/nu, 5–8 wk old females; CLEA Japan Inc., Tokyo, Japan) was performed as described previously [39] in strict accordance with the recommendations for the Handling of Laboratory Animals for Biomedical Research, as documented by the Institutional Animal Care and Use Committee (IACUC) at the Kinki University Faculty of Medicine (Permit Number: KAME-24-005). The stable transfectants of G361 cells expressing CYGB and control shRNAs were suspended in PBS and Matrigel (BD Biosciences) (1∶1) at a density of 1×10^7^ cells/ml. One million cells were injected subcutaneously into the interscapular region of mice (n = 5). Tumor size was assessed at 3 to 4-day interval and the tumor volume V was calculated according to the formula: V  =  W^2^×L×0.5, where W and L are tumor width and length, respectively. At the end of the experiment, the mice were killed and the xenografts were resected, fixed in 10% buffered formalin for 10 h, and processed for histological analysis. Apoptosis was determined by immunohistochemistry using anti-cleaved caspase 3 antibody (Cell Signaling Technology) as previously described [39].

### Cell Proliferation Assay

Cell proliferation was assessed using an MTT assay, as described previously [36].

### Measurement of ROS Production

Cells were transfected with CYGB siRNA or control siRNA and ROS generation was detected using 2′-, 7′-dichlorofluorescein diacetate (DCFH-DA) (Invitrogen). The cells were incubated with 10 μM of DCFH-DA for 30 min at 37°C and washed twice with PBS. After trypsinization, the cells were immediately analyzed using a FACScan flow cytometer (BD Biosciences, San Jose, CA).

### Assessment of Cell Death

Cell death was analyzed by staining the cells using an annexin V-FITC apoptosis detection kit I (BD Pharmingen, San Diego, CA), a procedure that reveals both apoptosis and necrosis. Briefly, G361 cells were transfected with CYGB siRNA or control siRNA and were treated with or without 100 μM H_2_O_2_ for 24 hours. After trypsinization followed by washing in PBS, the cells (1×10^5^) were resuspended in 100 μL binding buffer, to which FITC-Annexin and propidium iodide (PI) have been added, and incubated for 15 min. Cell death was measured using a flow cytometer.

### Statistical Analysis

The statistical analyses were performed using Microsoft Excel to calculate the SE and to test for statistically significant differences between the samples using the Student *t* test. A *P* value of <0.05 was considered statistically significant.

## Supporting Information

Figure S1 ***CYGB*** **mRNA is abundantly expressed in some melanoma cells.** **A.** Northern blot analysis of *CYGB* mRNA in A549, T47D, T98G and 5 melanoma cells under normoxic (N) and anoxic (0.1%–0.2% O_2_) (A) conditions. β-actin was used as a loading control. **B.** The relative CYGB expression levels that were assessed by realtime quantitative PCR using TaqMan probes and were compared with 18S rRNA expression are listed for the same cells as those analyzed in A. SEM values are shown in parenthesis.
(TIF)

Figure S2 **CYGB is predominantly distributed in melanocytes within the skin.** **A.** Immunoblot analysis of CYGB protein in NHDF, keratinocytes and melanocytes from skin and 8 melanoma cell lines (WM35 to HS294T). The image was obtained using ImageQuant LAS 3000 with an exposure time of 120 sec. **B.** Immunocytochemistry analysis of melanocytes, G361 and A375 cells using CYGB antibody. Scale bar, 5 μm.
(TIF)

Figure S3 ***CYGB*** **mRNA expression is reduced in most melanoma tissues during melanocyte-to-melanoma transition.** *CYGB* mRNA expression was compared in eight melanocyte cell lines (shown as red bars) and melanoma tissues from 82 patients deposited in the GSE29359 GEO dataset. The bar chart was drawn based on the meta-analysis described in the legend to Table S4. A horizontal line is drawn to show tissues with a relatively high expression of CYGB (higher than 0.3).
(TIF)

Figure S4 ***CYGB*** **mRNA expression is inversely correlated with the methylation status.** The GSE28356 GEO dataset with a platform of Illumina HumanMethylation27 BeadChip was meta-analyzed for the methylation status of the CYGB gene promoter (gene ID cg17040807) in 9 melanoma cell lines and one melanocyte pool, which was used as a normalization control. The beta value that indicated a continuous, quantitative measurement of DNA methylation, ranging from 0 (completely unmethylated) to 1 (completely methylated), was used for the calculation. The expression of cytoglobin mRNA was estimated using the GSE7152 dataset that analyzed 35 melanoma cell lines with an Affymetrix expression microarray platform. The probesets for cytoglobin mRNA (1553572_a_at) and GAPDH mRNA (M33197_M_at), a normalization control, were used. The nine cells analyzed in common to both datasets are listed **(A)** and compared for their normalized levels of the CYGB expression and *CYGB* promoter methylation **(B)**.
(TIF)

Figure S5 **Efficacy of shRNA-mediated CYGB knockdown in G361 cells.** G361 cells stably expressing shRNA against CYGB and control shRNA were generated by retrovirus transduction. **A.** Confirmation of CYGB knockdown in the cell line expressing CYGB shRNA at the protein level by Western analysis. β-actin was used as a loading control. **B.** Growth analysis of CYGB knockdown in G361 cells. G361 cells expressing CYGB shRNA and control shRNA were seeded in 96 well plates (2,000 cells/well) and cell growth was determined by MTT assay. OD value, 570 nm. bars, SEM. * *P*<0.05, ** *P*<0.01. **C.** Immunohistochemistry pictures of cleaved caspases 3-positive cells in G361 xenografts (Scale bar, 100 μm). **D.** Quantification of the apoptosis-signal in C (P<0.01, mean ± SD, n = 5).
(TIF)

Table S1 **Cell lines used in the experiment and their origins.** The list covers the cell lines analyzed by immunoblotting (Fig. 1) and northern blotting (Fig. 2*A*). The other cell lines subjected to preliminary screening using northern blotting included the following: H69, PC14, H1299, A427, Calu1, H520, H460, H1650, PC-9, H1975 (lung cancer), SKBR3, MDA-MB-468, MDA-MB-231, BT549, HCC1954 (breast cancer), Colo201, HCT116, WiDr, LoVo, SW480 (colon cancer), MKN1, IM95, MKN7, SNU1 (gastric cancer), LnCAP, Du145 (prostatic cancer), SCOV3, OVCAR3 (ovarian cancer), Caki-1, RCC4 (renal cell carcinoma), BxPC3, Capan1 (pancreatic cancer), A172, U87 (glioblastoma) and Hep3B (hepatoma), all of which gave no positive signal for CYGB.
(DOC)

Table S2 **Expression of cytoglobin mRNA in cell lines from several cancer types (1).** The GSE10843 GEO dataset with an Affymetrix expression microarray platform (GeneChip, HG-U133_Plus_2) was meta-analyzed for the expression of cytoglobin mRNA using 1553572_a_at, a transcript ID for cytoglobin. A transcript of glyceraldehyde 3-phosphate dehydrogenase (GAPDH; M33197_M_at) was used as a normalization control. To make the expression ratios comparable, the normalized CYGB/GAPDH value for MEWO melanoma cell line was set equal to 1. The database included 118 cell lines consisting of 15 melanoma (highlighted in red), 12 breast cancer, 50 lung cancer, 9 ovarian cancer, 6 lymphoma, and 26 colon cancer cell lines. The samples were aligned according to the order of the normalized expression level of *CYGB* mRNA.
(XLS)

Table S3 **Expression of cytoglobin mRNA in cell lines from several cancer types (2).** Several GEO datasets with an Affymetrix expression microarray platform (GeneChip) were used for the meta-analysis of cytoglobin mRNA expression (1553572_a_at) and a GAPDH mRNA (M33197_M_at) as a normalization control. To make the expression ratios comparable for all cell lines listed in both Tables S2 and S3, the normalized CYGB/GAPDH value for MEWO, which was set equal to 1 in Table S2, was introduced. The five datasets, GSE8332, GSE17714, GSE22563, GSE15455, and GSE9171, included 20 pancreatic, 11 neuroblastoma, 11 renal cell carcinoma, 33 gastric, and 17 glioblastoma cell lines, respectively. The samples with relatively abundant *CYGB* mRNA are highlighted.
(XLS)

Table S4 **Expression of cytoglobin mRNA in eight melanocyte cell lines and melanoma tissues from 79 patients.** The GSE29359 GEO dataset with an Ilumina expression microarray platform (beadarray) was meta-analyzed for the expression of cytoglobin mRNA using ILMN_1758128, a transcript ID for cytoglobin. A GAPDH transcript (ILMN_2038778) was used as a normalization control. The melanoma samples were aligned according to the order of the normalized expression level of CYGB mRNA. Three patients (62, 64 and 82) with poor GAPDH expression values were excluded from the comparison.
(DOC)
