# Supplementary figures and images for: Melanoma Transition Is Frequently Accompanied by a Loss of Cytoglobin Expression in Melanocytes: A Novel Expression Site of Cytoglobin
Source: PLoS One. 2014 Apr 10;9(4):e94772. doi: 10.1371/journal.pone.0094772 (PMC3983271; doi:10.1371/journal.pone.0094772)

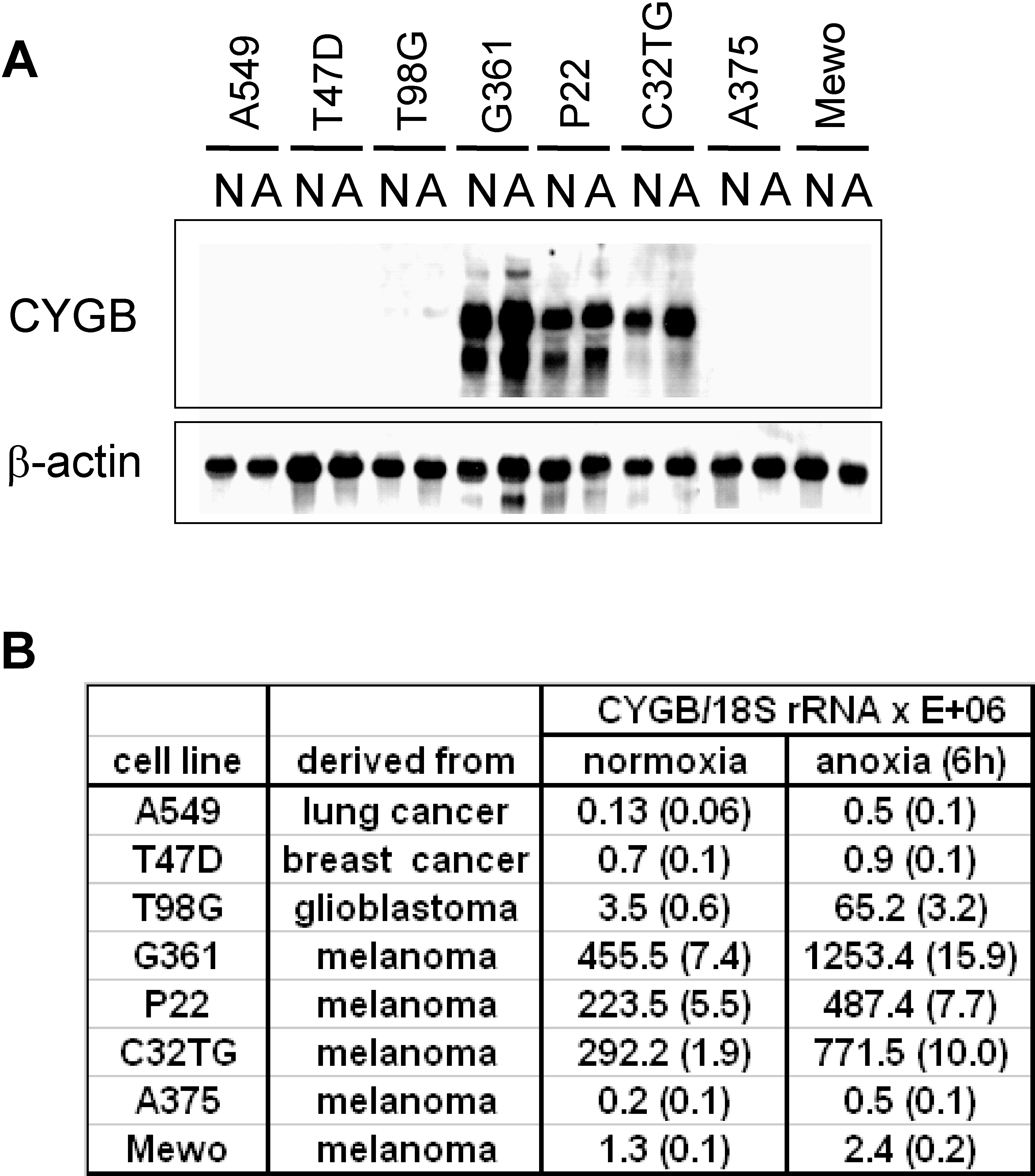

Supplement: Figure S1 — CYGB mRNA is abundantly expressed in some melanoma cells. A. Northern blot analysis of CYGB mRNA in A549, T47D, T98G and 5 melanoma cells under normoxic (N) and anoxic (0.1%–0.2% O2) (A) conditions. β-actin was used as a loading control. B. The relative CYGB expression levels that were assessed by realtime quantitative PCR using TaqMan probes and were compared with 18S rRNA expression are listed for the same cells as those analyzed in A. SEM values are shown in parenthesis. (TIF) [file pone.0094772.s001.tif]

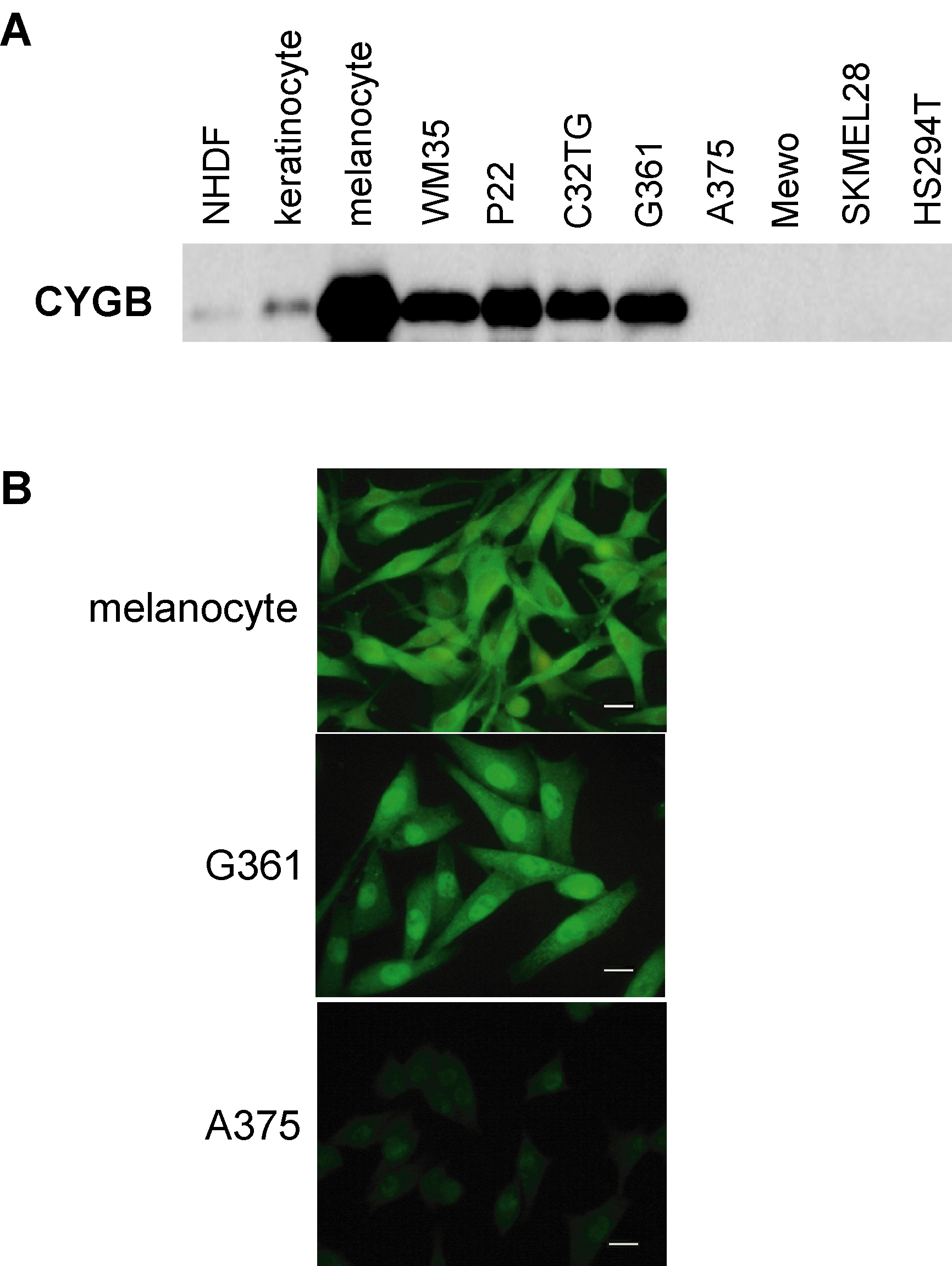

Supplement: Figure S2 — CYGB is predominantly distributed in melanocytes within the skin. A. Immunoblot analysis of CYGB protein in NHDF, keratinocytes and melanocytes from skin and 8 melanoma cell lines (WM35 to HS294T). The image was obtained using ImageQuant LAS 3000 with an exposure time of 120 sec. B. Immunocytochemistry analysis of melanocytes, G361 and A375 cells using CYGB antibody. Scale bar, 5 μm. (TIF) [file pone.0094772.s002.tif]

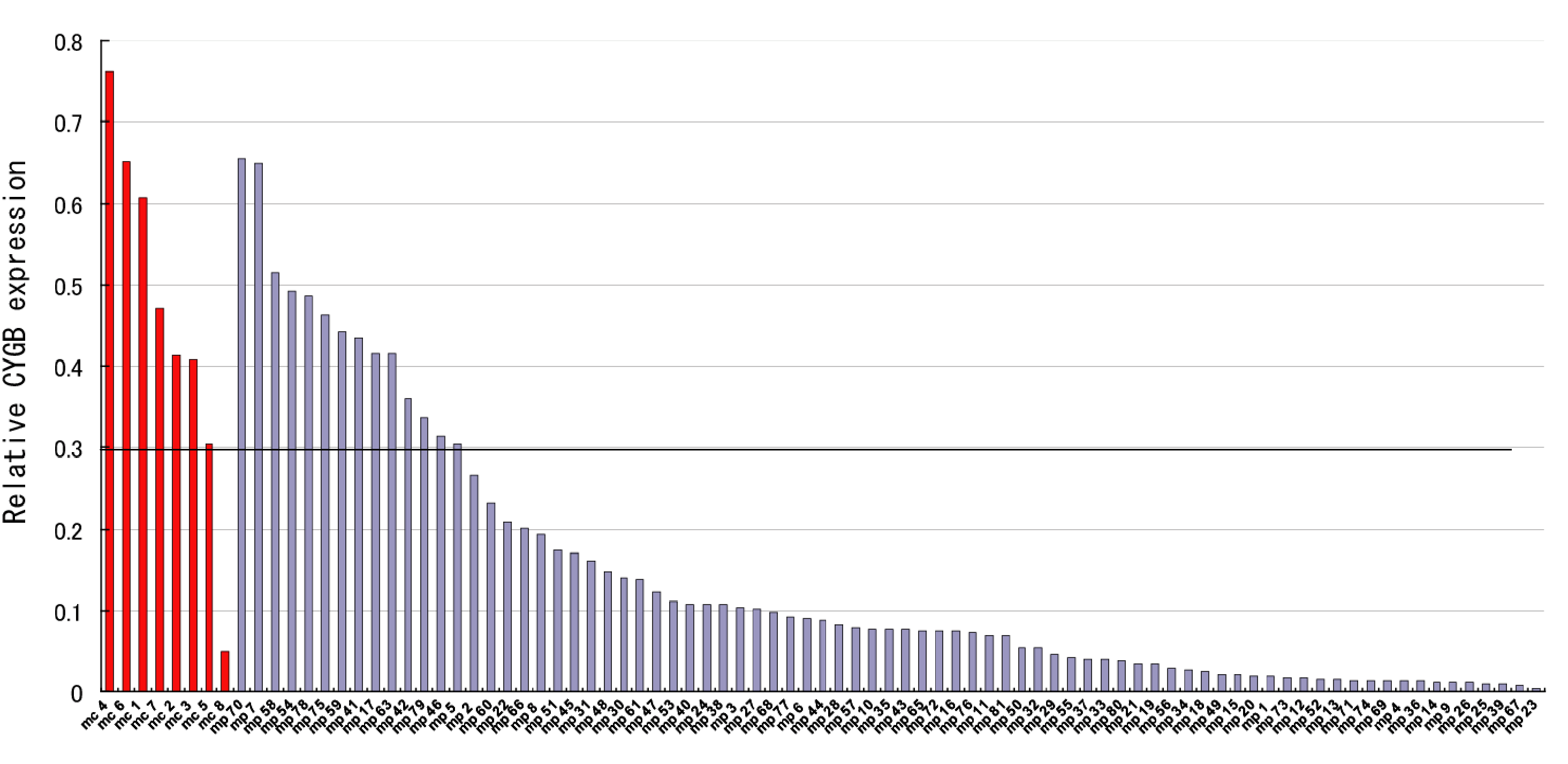

Supplement: Figure S3 — CYGB mRNA expression is reduced in most melanoma tissues during melanocyte-to-melanoma transition. CYGB mRNA expression was compared in eight melanocyte cell lines (shown as red bars) and melanoma tissues from 82 patients deposited in the GSE29359 GEO dataset. The bar chart was drawn based on the meta-analysis described in the legend to Table S4. A horizontal line is drawn to show tissues with a relatively high expression of CYGB (higher than 0.3). (TIF) [file pone.0094772.s003.tif]

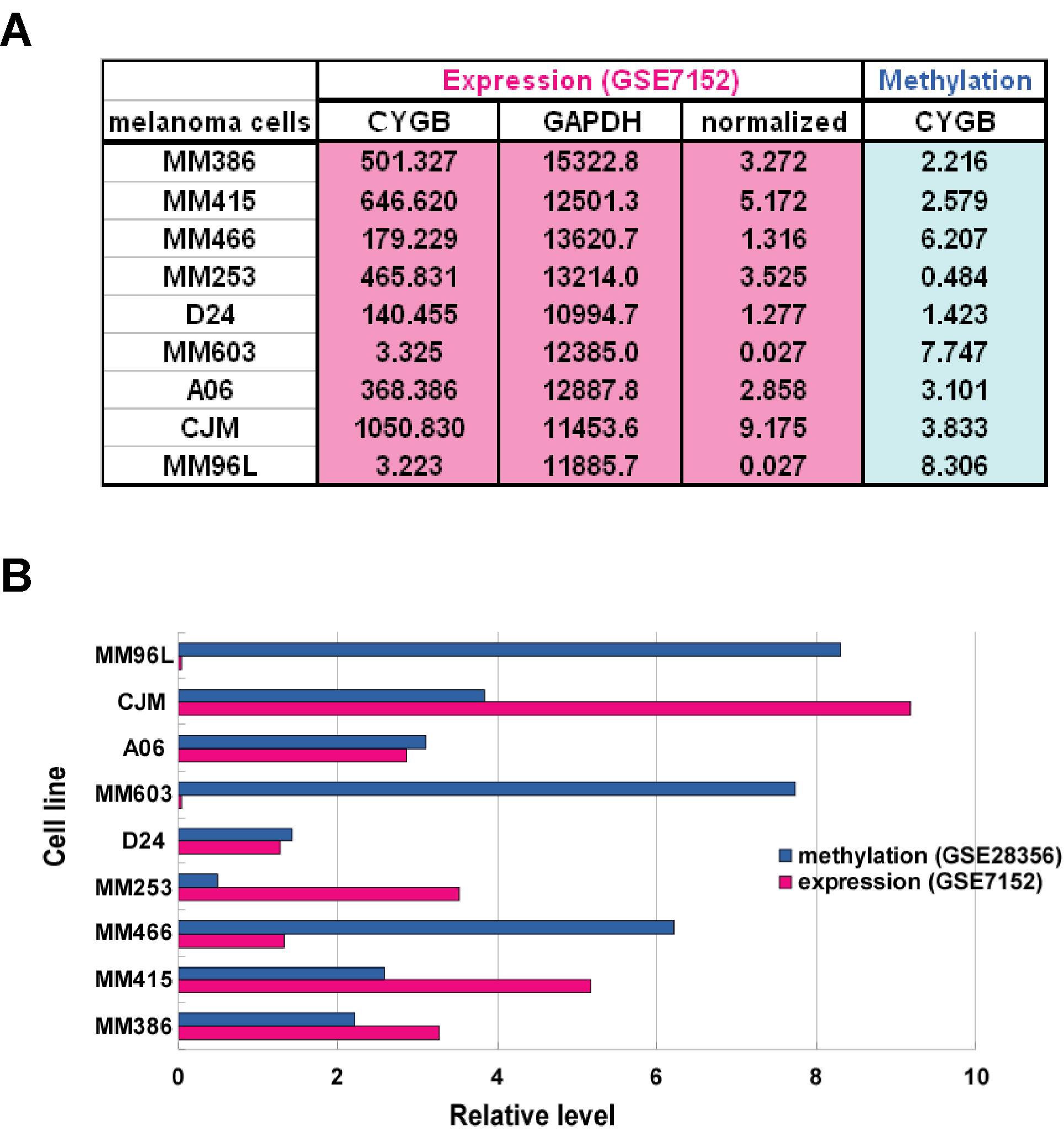

Supplement: Figure S4 — CYGB mRNA expression is inversely correlated with the methylation status. The GSE28356 GEO dataset with a platform of Illumina HumanMethylation27 BeadChip was meta-analyzed for the methylation status of the CYGB gene promoter (gene ID cg17040807) in 9 melanoma cell lines and one melanocyte pool, which was used as a normalization control. The beta value that indicated a continuous, quantitative measurement of DNA methylation, ranging from 0 (completely unmethylated) to 1 (completely methylated), was used for the calculation. The expression of cytoglobin mRNA was estimated using the GSE7152 dataset that analyzed 35 melanoma cell lines with an Affymetrix expression microarray platform. The probesets for cytoglobin mRNA (1553572_a_at) and GAPDH mRNA (M33197_M_at), a normalization control, were used. The nine cells analyzed in common to both datasets are listed (A) and compared for their normalized levels of the CYGB expression and CYGB promoter methylation (B). (TIF) [file pone.0094772.s004.tif]

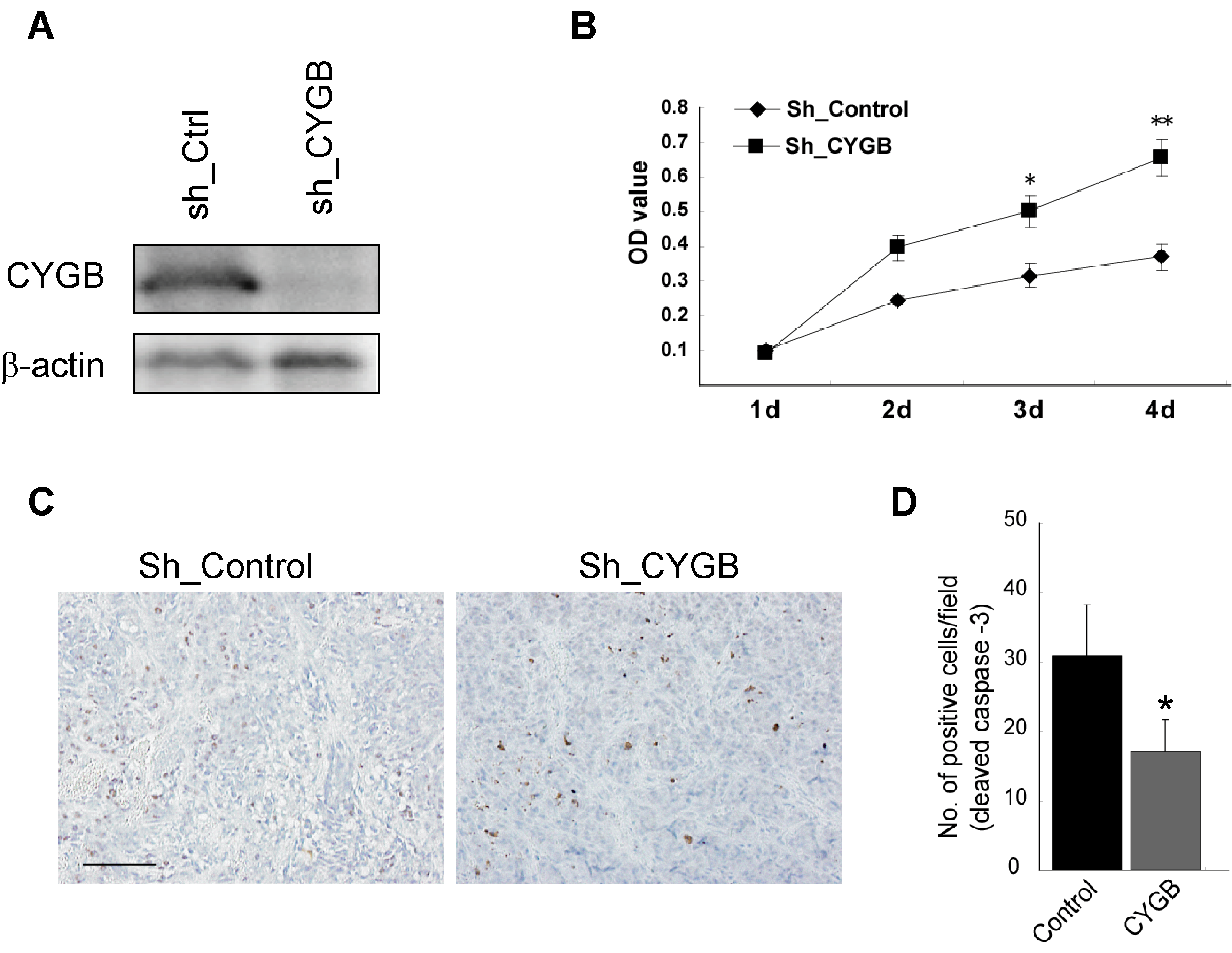

Supplement: Figure S5 — Efficacy of shRNA-mediated CYGB knockdown in G361 cells. G361 cells stably expressing shRNA against CYGB and control shRNA were generated by retrovirus transduction. A. Confirmation of CYGB knockdown in the cell line expressing CYGB shRNA at the protein level by Western analysis. β-actin was used as a loading control. B. Growth analysis of CYGB knockdown in G361 cells. G361 cells expressing CYGB shRNA and control shRNA were seeded in 96 well plates (2,000 cells/well) and cell growth was determined by MTT assay. OD value, 570 nm. bars, SEM. * P<0.05, ** P<0.01. C. Immunohistochemistry pictures of cleaved caspases 3-positive cells in G361 xenografts (Scale bar, 100 μm). D. Quantification of the apoptosis-signal in C (P<0.01, mean ± SD, n = 5). (TIF) [file pone.0094772.s005.tif]
