# Supplementary material for: Melanoma Transition Is Frequently Accompanied by a Loss of Cytoglobin Expression in Melanocytes: A Novel Expression Site of Cytoglobin
Source: PLoS One. 2014 Apr 10;9(4):e94772. doi: 10.1371/journal.pone.0094772 (PMC3983271; doi:10.1371/journal.pone.0094772)
Supplement: Table S1 — Cell lines used in the experiment and their origins. The list covers the cell lines analyzed by immunoblotting (Fig. 1) and northern blotting (Fig. 2A ). The other cell lines subjected to preliminary screening using northern blotting included the following: H69, PC14, H1299, A427, Calu1, H520, H460, H1650, PC-9, H1975 (lung cancer), SKBR3, MDA-MB-468, MDA-MB-231, BT549, HCC1954 (breast cancer), Colo201, HCT116, WiDr, LoVo, SW480 (colon cancer), MKN1, IM95, MKN7, SNU1 (gastric cancer), LnCAP, Du145 (prostatic cancer), SCOV3, OVCAR3 (ovarian cancer), Caki-1, RCC4 (renal cell carcinoma), BxPC3, Capan1 (pancreatic cancer), A172, U87 (glioblastoma) and Hep3B (hepatoma), all of which gave no positive signal for CYGB. (DOC) [file pone.0094772.s006.doc]

**Table S1 Cell lines used in the experiment and their origins.**

| **Cell line** | **Origin** |
| --- | --- |
| **G361** | **Melanoma** |
| **P22** | **Melanoma** |
| **C32TG** | **Melanoma** |
| **WM35** | **Melanoma** |
| **A375** | **Melanoma** |
| **Mewo** | **Melanoma** |
| **SKMEL28** | **Melanoma** |
| **HS294T** | **Melanoma** |
| **A549** | **Lung adenocarcinoma** |
| **SBC3** | **Lung, small cell carcinoma** |
| **MCF7** | **Breast cancer** |
| **T47D** | **Breast cancer** |
| **T98G** | **Glioblastoma** |
| **AZ521** | **Gastric cancer** |
| **DLD-1** | **Colon cancer** |
| **SCH** | **Choriocarcinoma** |
| **Hela** | **Uterine cancer** |
| **PC3** | **Prostatic cancer** |
| **HepG2** | **Hepatoma** |
| **MIA PaCa-2** | **pancreatic cancer** |

The list covers the tumor cell lines analyzed by realtime quantitative PCR (Figure 1), immunoblotting (Figure 2A) or northern blotting (Supplementary Figure S1). The other cell lines subjected to preliminary screening by northern blotting include: H69, PC14, H1299, A427, Calu1, H520, H460, H1650, PC-9, H1975 (lung cancer), SKBR3, MDA-MB-468, MDA-MB-231, BT549, HCC1954 (breast cancer), Colo201, HCT116, WiDr, LoVo, SW480 (colon cancer), MKN1, IM95, MKN7, SNU1 (gastric cancer), LnCAP, Du145 (prostatic cancer), SCOV3, OVCAR3 (ovarian cancer), Caki-1, RCC4 (renal cell carcinoma), BxPC3, Capan1 (pancreatic cancer), A172, U87 (glioblastoma), Hep3B (hepatoma), all of which gave no positive signal for CYGB.
