# Supplementary material for: Melanoma Transition Is Frequently Accompanied by a Loss of Cytoglobin Expression in Melanocytes: A Novel Expression Site of Cytoglobin
Source: PLoS One. 2014 Apr 10;9(4):e94772. doi: 10.1371/journal.pone.0094772 (PMC3983271; doi:10.1371/journal.pone.0094772)
Supplement: Table S4 — Expression of cytoglobin mRNA in eight melanocyte cell lines and melanoma tissues from 79 patients. The GSE29359 GEO dataset with an Ilumina expression microarray platform (beadarray) was meta-analyzed for the expression of cytoglobin mRNA using ILMN_1758128, a transcript ID for cytoglobin. A GAPDH transcript (ILMN_2038778) was used as a normalization control. The melanoma samples were aligned according to the order of the normalized expression level of CYGB mRNA. Three patients (62, 64 and 82) with poor GAPDH expression values were excluded from the comparison. (DOC) [file pone.0094772.s009.doc]

**Table S4 Expression of cytoglobin mRNA in eight melanocyte cell lines and melanoma tissues from 79 patients.**

|  |  | **GAPDH** | **CYG** | **CYG/GAPDH** |
| --- | --- | --- | --- | --- |
| **GEO samples** | **biopsies** | **ID: 2038778** | **ID: 1758128** |  |
| GSM725734 | normal melanocyte 1 | 6383 | 3872 | 0.606611311 |
| GSM725735 | normal melanocyte 2 | 8400 | 3473 | 0.413452381 |
| GSM725736 | normal melanocyte 3 | 10030 | 4091 | 0.407876371 |
| GSM725737 | normal melanocyte 4 | 6343 | 4831 | 0.76162699 |
| GSM725738 | normal melanocyte 5 | 6347 | 1937 | 0.305183551 |
| GSM725739 | normal melanocyte 6 | 6926 | 4501 | 0.649870055 |
| GSM725740 | normal melanocyte 7 | 7830 | 3691 | 0.471392082 |
| GSM725741 | normal melanocyte 8 | 17369 | 880 | 0.050664978 |
| GSM725811 | melanoma patient 70 | 5708 | 3735 | 0.654344779 |
| GSM725748 | melanoma patient 7 | 12082 | 7830 | 0.648071511 |
| GSM725799 | melanoma patient 58 | 9937 | 5111 | 0.514340344 |
| GSM725795 | melanoma patient 54 | 2646 | 1301 | 0.491685563 |
| GSM725819 | melanoma patient 78 | 22454 | 10928 | 0.486683887 |
| GSM725816 | melanoma patient 75 | 22211 | 10301 | 0.463779209 |
| GSM725800 | melanoma patient 59 | 5534 | 2450 | 0.442717745 |
| GSM725782 | melanoma patient 41 | 1316 | 572 | 0.434650456 |
| GSM725758 | melanoma patient 17 | 8215 | 3419 | 0.416189897 |
| GSM725804 | melanoma patient 63 | 4307 | 1786 | 0.414673787 |
| GSM725783 | melanoma patient 42 | 5376 | 1938 | 0.360491071 |
| GSM725820 | melanoma patient 79 | 18193 | 6142 | 0.337602375 |
| GSM725787 | melanoma patient 46 | 10897 | 3422 | 0.314031385 |
| GSM725746 | melanoma patient 5 | 6347 | 1937 | 0.305183551 |
| GSM725743 | melanoma patient 2 | 7586 | 2017 | 0.265884524 |
| GSM725801 | melanoma patient 60 | 9331 | 2156 | 0.231057764 |
| GSM725763 | melanoma patient 22 | 5668 | 1184 | 0.208892025 |
| GSM725807 | melanoma patient 66 | 9836 | 1975 | 0.200793005 |
| GSM725749 | melanoma patient 8 | 9845 | 1898 | 0.192788217 |
| GSM725792 | melanoma patient 51 | 7689 | 1340 | 0.174274938 |
| GSM725786 | melanoma patient 45 | 7943 | 1349 | 0.169835075 |
| GSM725772 | melanoma patient 31 | 12965 | 2076 | 0.160123409 |
| GSM725789 | melanoma patient 48 | 18218 | 2680 | 0.147107257 |
| GSM725771 | melanoma patient 30 | 12172 | 1692 | 0.139007558 |
| GSM725802 | melanoma patient 61 | 7991 | 1094 | 0.136904017 |
| GSM725788 | melanoma patient 47 | 12269 | 1501 | 0.122340859 |
| GSM725794 | melanoma patient 53 | 7050 | 789 | 0.111914894 |
| GSM725781 | melanoma patient 40 | 6898 | 740 | 0.107277472 |
| GSM725765 | melanoma patient 24 | 10101 | 1083 | 0.107217107 |
| GSM725779 | melanoma patient 38 | 15912 | 1703 | 0.107026144 |
| GSM725744 | melanoma patient 3 | 9771 | 1003 | 0.102650701 |
| GSM725768 | melanoma patient 27 | 19812 | 2009 | 0.10140319 |
| GSM725809 | melanoma patient 68 | 11576 | 1132 | 0.097788528 |
| GSM725818 | melanoma patient 77 | 17419 | 1591 | 0.091337046 |
| GSM725747 | melanoma patient 6 | 10030 | 902 | 0.089930209 |
| GSM725785 | melanoma patient 44 | 21635 | 1923 | 0.088883753 |
| GSM725769 | melanoma patient 28 | 11982 | 976 | 0.081455517 |
| GSM725798 | melanoma patient 57 | 19703 | 1533 | 0.07780541 |
| GSM725751 | melanoma patient 10 | 17638 | 1367 | 0.077503118 |
| GSM725776 | melanoma patient 35 | 19454 | 1503 | 0.077259175 |
| GSM725784 | melanoma patient 43 | 10252 | 792 | 0.077253219 |
| GSM725806 | melanoma patient 65 | 5362 | 399 | 0.074412533 |
| GSM725813 | melanoma patient 72 | 16970 | 1259 | 0.074189747 |
| GSM725757 | melanoma patient 16 | 10678 | 788 | 0.073796591 |
| GSM725817 | melanoma patient 76 | 10831 | 786 | 0.072569477 |
| GSM725752 | melanoma patient 11 | 18974 | 1322 | 0.069674291 |
| GSM725822 | melanoma patient 81 | 15667 | 1080 | 0.068934704 |
| GSM725791 | melanoma patient 50 | 13214 | 710 | 0.053730891 |
| GSM725773 | melanoma patient 32 | 9530 | 507 | 0.05320042 |
| GSM725770 | melanoma patient 29 | 11117 | 509 | 0.045785734 |
| GSM725796 | melanoma patient 55 | 8794 | 369 | 0.041960428 |
| GSM725778 | melanoma patient 37 | 17792 | 732 | 0.041142086 |
| GSM725774 | melanoma patient 33 | 9453 | 388 | 0.041045171 |
| GSM725821 | melanoma patient 80 | 30501 | 1178 | 0.038621685 |
| GSM725762 | melanoma patient 21 | 14235 | 495 | 0.034773446 |
| GSM725760 | melanoma patient 19 | 12587 | 424 | 0.033685549 |
| GSM725797 | melanoma patient 56 | 15223 | 429 | 0.028181042 |
| GSM725775 | melanoma patient 34 | 11955 | 323 | 0.027017984 |
| GSM725759 | melanoma patient 18 | 16527 | 396 | 0.023960791 |
| GSM725790 | melanoma patient 49 | 16366 | 342 | 0.020896982 |
| GSM725756 | melanoma patient 15 | 17102 | 357 | 0.020874751 |
| GSM725761 | melanoma patient 20 | 20005 | 392 | 0.019595101 |
| GSM725742 | melanoma patient 1 | 9795 | 190 | 0.019397652 |
| GSM725814 | melanoma patient 73 | 24722 | 438 | 0.017717013 |
| GSM725753 | melanoma patient 12 | 12972 | 220 | 0.016959605 |
| GSM725793 | melanoma patient 52 | 12455 | 196 | 0.015736652 |
| GSM725754 | melanoma patient 13 | 11531 | 170 | 0.014742867 |
| GSM725812 | melanoma patient 71 | 23549 | 329 | 0.013970869 |
| GSM725815 | melanoma patient 74 | 22414 | 308 | 0.013741412 |
| GSM725810 | melanoma patient 69 | 20924 | 284 | 0.013572931 |
| GSM725745 | melanoma patient 4 | 11994 | 153 | 0.012756378 |
| GSM725777 | melanoma patient 36 | 7747 | 97 | 0.012520976 |
| GSM725755 | melanoma patient 14 | 16136 | 199 | 0.012332672 |
| GSM725750 | melanoma patient 9 | 17520 | 216 | 0.012328767 |
| GSM725767 | melanoma patient 26 | 13309 | 161 | 0.012097077 |
| GSM725766 | melanoma patient 25 | 16951 | 175 | 0.010323875 |
| GSM725780 | melanoma patient 39 | 17068 | 156 | 0.009139911 |
| GSM725808 | melanoma patient 67 | 18661 | 134 | 0.007180751 |
| GSM725764 | melanoma patient 23 | 19916 | 84 | 0.004217714 |
| GSM725803 | melanoma patient 62 | 151* | 1940 | (-), *pvalue:0.2485876 |
| GSM725805 | melanoma patient 64 | 403** | 1188 | (-),**pvalue:0.09887005 |
| GSM725823 | melanoma patient 82 | 971*** | 1300 | (-),  ***pvalue:0.004237288 |

GSE29359 GEO dataset with a platform of Ilumina expression microarray (beadarray) was meta-analyzed for the expression of cytoglobin mRNA using ILMN_1758128, a transcript ID for cytoglobin. A GAPDH transcript (ILMN_2038778) was used as a normalization control. The melanoma samples were aligned according to the order of the normalized expression level of CYGB mRNA. Three patients (62, 64 and 82) having poor GAPDH expression values were excluded from comparison.
